# From lipid function to dysfunction: Very long-chain fatty acids as emerging regulators of neuroinflammatory pathways

**DOI:** 10.1016/j.isci.2026.114724

**Published:** 2026-01-16

**Authors:** Ranjan Kumar Sahu, Yunseon Yang, Hyung-Lok Chung

**Affiliations:** 1 Department of Neurology, Houston Methodist Research Institute, Houston, TX, USA; 2 Department of Neurology, Weill Cornell Medical College, New York, NY, USA

**Keywords:** natural sciences, biological sciences, biochemistry

## Abstract

Lipids are key structural and functional components of the brain, essential for cellular integrity and homeostasis. They regulate signaling pathways driving neuroinflammation, a key factor in many neurological disorders. We review the interplay between lipid metabolism and neuroinflammation, with a focus on very long-chain fatty acids, that are crucial for membrane integrity and of myelin formation. Insights from human genetic disorders highlight how lipid metabolic dysfunction triggers neuroinflammatory cascade. Studies in *Drosophila* now complement these findings by enabling rapid dissection of conserved pathways and testing of therapeutic strategies, including enzyme modulators, dietary interventions, and gene therapies. By bridging patient-derived insights with powerful *in vivo* modeling, *Drosophila* research is uncovering actionable mechanisms linking lipid dysregulation to neurodevelopmental and neurodegenerative disease.

## Lipids metabolism is crucial for CNS homeostasis

Lipids are crucial components of the brain, comprising a significant proportion of its structure and playing central roles in preserving structural integrity. They provide energy to various cells including neurons and glia via oxidation and formation of ketone bodies and contribute to cell signaling and myelin formation.

Under normal conditions, neurons primarily rely on glucose oxidation for ATP generation, because β-oxidation of fatty acids increases the risk of hypoxia by generating superoxide and consequent oxidative stress. To avoid toxicity from free fatty acids, neurons release them via apolipoprotein particles, which then enter astrocytes.^1^ Within astrocytes, free fatty acids are incorporated into lipid droplets (LDs) and utilized in mitochondrial β-oxidation.^1^ Astrocytes also synthesize lipids and transfer them to neurons to support neuronal survival and functions. However, if LDs and fatty acids accumulate in astrocytes, it stimulates neuronal β-oxidation, resulting in oxidative stress and formation of reactive microglia.^2^^,^^3^

Abnormalities in lipid metabolism, including very long-chain fatty acids (VLCFAs),^4^^,^^5^^,^^6^^,^^7^ LDs,^8^^,^^9^ and plasmalogens,^10^ significantly impact both neuronal and glial cells, resulting in pronounced neuroinflammation. Endoplasmic reticulum (ER), the primary site of lipid metabolism, contains many enzymes responsible for the metabolism of glycerolipids, sphingolipids, and sterols. Reports suggest the unfolded protein response (UPR), a signaling pathway that monitors ER homeostasis, plays a vital role in maintaining lipid and metabolic homeostasis. Excess mitochondrial and peroxisomal fatty acid β-oxidation induce reactive oxygen species (ROS) production in neural progenitor cells,^3^ and glial cells including microglia, astrocytes,^3^ and oligodendrocytes,^3^ resulting in lipotoxicity associated with ER stress and mitochondrial dysfunction. Elevated ROS can damage not only lipids in the cell membrane and activate the immune system but also mitochondrial components. Accumulation of LDs in microglia impairs the phagocytic function and increases ROS and proinflammatory cytokine secretion.^9^ When oxidative phosphorylation for fatty acid degradation in astrocytes is overwhelmed by excessive fatty acids, elevated acetyl-CoA levels induce astrocyte reactivity.^2^

**Figure 1:**
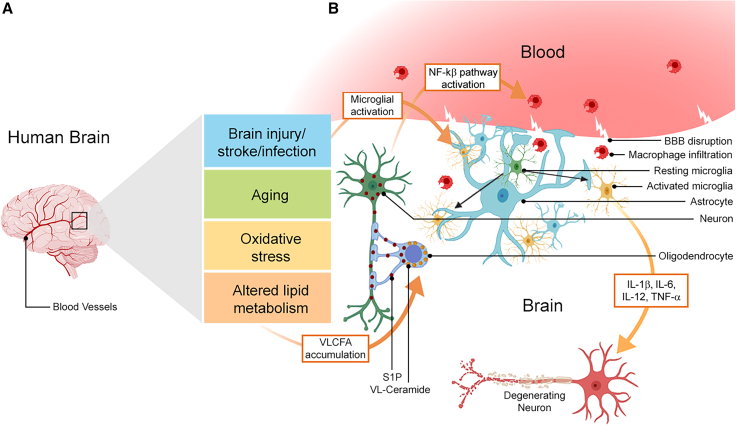
Vertebrate neuro-glial interactions and the BBB in neuroinflammation (A) Human adult brain highlights the blood vessels. (B) Cross-section of the human brain and factors inducing neuroinflammation. PAMPs and DAMPs are produced upon pathogen invasion and trauma, respectively, which often lead to microglial activation. The proinflammatory cytokines IL-1β, IL-6, IL-12, and TNF-α released from the activated microglia are often linked to BBB disruption. These molecular patterns cause astroglia proliferation, activation (astrogliosis), and dysfunction, leading to endothelial cell injury, BBB disruption, macrophage infiltration, and consequent neuronal death. Aging, oxidative stress, and altered lipid metabolism leads to the accumulation of VLCFAs in glia. Mutations in *ACOX1* lead to deposition and conversion of VL-ceramide (yellow) into S1P (maroon) and its transport to neurons to activate NF-κB signaling and macrophage recruitment. This figure was created with BioRender.

Other specific lipid metabolism pathways are implicated in central nervous system (CNS) pathobiology. Glial loss of ACOX1, which is involved in peroxisomal β-oxidation, can increase levels of sphingosine 1-phosphate (S1P) due to the accumulation of VLCFAs in *Drosophila.*^6^ Cellular stress can activate NOD1/2, which leads to inflammatory responses and NF-κB activation in mice via S1P (see Figure 1). Excess S1P is secreted by glial cells and absorbed by neurons, ultimately leading to neuroinflammation.^6^ Peroxisome plays a vital role in the catabolism of VLCFAs and ROS reduction, so dysfunctional peroxisomal β-oxidation can cause accumulation of VLCFAs and consequent pathology. For example, *ABCD1* encodes a peroxisomal transporter of VLCFAs in peroxisomes for their degradation via β-oxidation, and *ABCD1* mutations can cause VLCFA accumulation in tissues and plasma, potentially increasing ROS production.^11^^,^^12^

## Neuroinflammation and its association with neurodegeneration

Neuroinflammation is an inflammation within the brain and spinal cord, forming the CNS. The neuroinflammatory response is complex and multifaceted and includes defense system activation, recruitment of peripheral immune cells, and the release of protective and reparative chemicals.^13^^,^^14^ Neuroinflammation is a fundamental and conserved mechanism, occurring in many organisms including *Drosophila*,^15^^,^^16^ mice,^17^^,^^18^ and humans.

**Figure 2:**
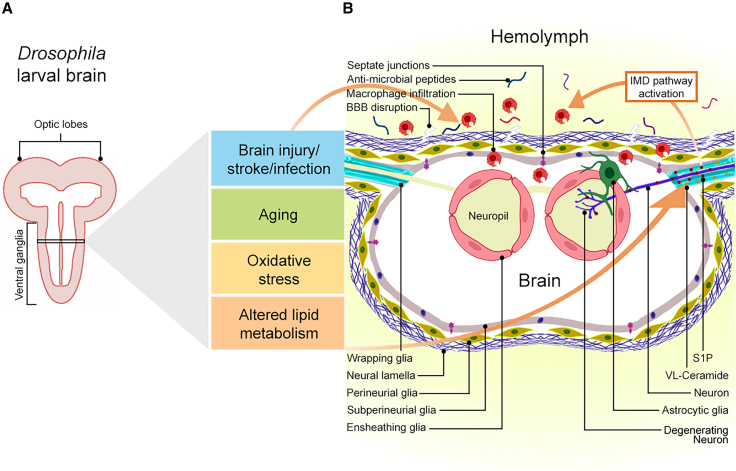
*Drosophila* neuro-glial interactions and the BBB in neuroinflammation (A) The *Drosophila* larval CNS has two brain lobes and ventral ganglia. (B) Cross-section of the ventral ganglia and factors inducing neuroinflammation. The CNS is surrounded by hemolymph. The glial populations forming the BBB are the neural lamella, perineurial glia, and sub-perineurial glia (forms intracellular septate junctions for selective transport of materials from hemolymph). Ensheathing glia encapsulates the neuropil (the synaptic space), while astrocytes project into the neuropil from the BBB to interact with neurons. Wrapping glia encapsulates axons of peripheral neurons. Several factors including brain injury, aging, oxidative stress, and altered lipid metabolism leads to neuroinflammation, which is characterized by VLCFA accumulation. Mutations in *ACOX1* lead to deposition and conversion of VL-ceramide (yellow) into S1P (maroon) in wrapping glia and transport to neurons. S1P activates the IMD pathway, leading to the production of anti-microbial peptides and macrophage infiltration. This figure was created with BioRender.

Several factors can cause or contribute to neuroinflammation including physical brain injury, microbial infections,^19^ exposure to harmful chemicals, chronic physiological and oxidative stress,^20^ autoimmune diseases,^21^ aging and, in some cases, genetic factors^13^^,^^22^ (see Figures 1 and 2). Microglia detect alterations and maintain homeostasis within the brain environment. They are also crucial for sustaining myelin integrity and synaptic remodeling.^23^^,^^24^ Similarly, astrocytes, the most abundant glial cells in CNS, support neuronal function and maintain brain homeostasis. They are involved in the blood-brain barrier (BBB) formation in vertebrates (see Figure 1), provide nutrition to neurons, modulate synapses, and clear debris.^25^^,^^26^ Both microglia and astrocytes become activated in response to brain or neuronal injury.^27^ Activated microglia and reactive astrocytes release cytokines (see Figures 1 and 2), which can exert beneficial and detrimental effects on the brain environment. For example, several neurotrophic factors are upregulated in these neuroprotective glial cell types, including brain-derived neurotrophic factor (BDNF), glial cell line-derived neurotrophic factor (GDNF),^28^^,^^29^ and other anti-inflammatory cytokines that promote the release of transforming growth factor (TGF)-β, interleukin (IL)-4, and IL-10.^26^ In contrast, glial cells produce lactosylceramide during chronic inflammation that may lead to neurodegeneration or upregulate complement cascade genes to induce damaging tumor necrosis factor (TNF)-α, IL-1β, and nitric oxide (NO).^29^ Harmful signaling pathways in reactive astrocytes can also be induced by various factors including sphingolipids (sphingosine 1-phosphate [S1P] and lactosylceramide) and neurotrophins.^25^

Therefore, while neuroinflammation serves as a protective mechanism for the nervous system, prolonged neuroinflammation can cause neurotoxicity and is strongly associated with neurodegenerative processes.^24^ In this way, chronic microglial activation coupled with persistent production of proinflammatory cytokines contribute to serious neurodegenerative disorders such as Alzheimer’s disease (AD), Parkinson’s disease (PD), Huntington’s disease (HD), amyotrophic lateral sclerosis (ALS), and multiple sclerosis (MS). Given the explicit correlation between neurodegenerative diseases and neuroinflammation, the analysis of the underlying pathways is essential for understanding pathoetiology and advancing therapeutic interventions for many neurological diseases.

In this review, we focus on lipid metabolism, particularly VLCFAs, as a cause of neuroinflammation. We introduce various mechanisms by which lipid metabolism influences neuronal health, highlighting how the *Drosophila* model can be exploited for analyzing human diseases.

## Role of VLCFAs in brain health and disease

Fatty acids are the primary building blocks of most lipids, often esterified with alcohol moieties such as glycerol or sphingosine. They are categorized into four major groups based on chain length: short-chain fatty acids (SCFAs; <6 carbons), medium-chain fatty acids (MCFAs; 6–12 carbons), long-chain fatty acids (LCFAs; 13–21 carbon), and VLCFAs (>21 carbons). Compared with other groups of fatty acids, VLCFAs are present at relatively low concentrations within biological systems^30^ but nevertheless act as important components of cells such as lipid rafts and myelin, where they play crucial roles in physiological processes.^6^^,^^31^ VLCFAs are primarily esterified in sphingolipids, a class of essential lipids for cell membrane structure and signaling, including sphingomyelin, ceramides, gangliosides, sulfatides, and galactocerebrosides.^5^^,^^6^^,^^31^^,^^32^ Therefore, sphingolipids such as ceramides often contain saturated and monounsaturated VLCFAs that play crucial roles in neuronal polarity and various diseases.^6^^,^^20^^,^^30^^,^^31^^,^^33^^,^^34^^,^^35^

VLCFAs are degraded in peroxisomes, specialized cellular organelles that play a role in lipid metabolism, including lipid breakdown and detoxification (see Box 2). They are crucial for the formation of various lipids, such as sphingolipids and ceramides, which are essential for the integrity of cell membrane. Despite their low abundance, VLCFAs constitute a disproportionately large fraction of myelin lipids compared with other plasma membranes.^4^^,^^5^^,^^6^^,^^35^^,^^36^^,^^37^

Box 1 **Neurological disorders associated with VLCFA metabolism**

*AD and PD*

Several reports throughout the last decade have implicated lipid metabolism in neurodegenerative diseases like AD^48^^,^^108^^,^^109^ and PD.^110^^,^^111^ Several mechanisms, including abnormalities in lipid metabolism, have been proposed to explain the origins of neuroinflammation in AD.^112^^,^^113^ For example, VLCFAs have been shown to accumulate in the brains of AD patients, where they were associated with neurofibrillary tangles. VLCFAs rapidly promote synuclein clustering and make it more prone to forming harmful aggregates^114^ and impaired VLCFA metabolism increases α-synuclein toxicity in a yeast model of PD.^115^

*Peroxisome biogenesis disorders*

Peroxisome biogenesis disorders are rare genetic conditions characterized by the absence of functionally competent peroxisomes. Mutations in any of the peroxisome assembly protein peroxin (*PEX*) genes^116^^,^^117^ disrupt peroxisomal function and lead to impaired β-oxidation of VLCFAs, thereby increasing VLCFA levels in tissues.^118^ Affected individuals may exhibit hypotonia, intellectual disabilities, facial abnormalities, and sometimes seizures^117^ and possess elevated VLCFA levels in the brain,^119^ which could be a potential cause of neuroinflammation.

*X-ALD*

X-ALD, which mainly affects the brain and adrenal glands, results from mutations in *ABCD1* and is characterized by VLCFA accumulation due to defective peroxisomal β-oxidation and adrenal insufficiency.^44^^,^^120^ Cerebral X-ALD particularly manifests in children (4–10 years) and is characterized by neuroinflammation, cerebral demyelination, and potential life-threatening complications. In patients with cerebral X-ALD, VLCFAs stimulate the cell membrane of macrophages to promote a proinflammatory response.^35^ Adrenomyeloneuropathy, a milder form of X-ALD, is characterized by its adult-onset (20–40 years), and it primarily affects the spinal cord and PNS to cause muscular stiffness, sensory ataxia, and spastic paraparesis.^121^^,^^122^

*Sjogren-Larsson syndrome*

Sjogren-Larsson syndrome is an autosomal recessive disorder caused by mutations in *ALDH3A2*,^123^^,^^124^ the product of which impairs the breakdown of fatty aldehydes derived from VLCFAs. Accumulation of fatty aldehydes leads to neurological symptoms such as intellectual disability, spasticity, and ichthyosis.^123^^,^^124^

*Mitchell syndrome*

Mitchell syndrome is a rare autosomal dominant genetic disorder first reported in 2020 characterized by episodic demyelination,^20^ and gait instability caused by progressive loss of both sensory and motor neurons together with hearing loss.^20^^,^^125^^,^^126^ Mitchell syndrome is caused by a heterozygous gain-of-function mutation (pN237S) in *ACOX1*, the rate limiting gene in the β-oxidation pathway. Phenotypes of patients with *ACOX1* gain-of-function mutations contrast sharply with patients with ACOX1 deficiency, as VLCLA levels in Mitchell syndrome are unchanged but ROS levels increase to cause neuroinflammation.^20^

Box 2 **VLCFA metabolism**

*VLCFA synthesis*

Neuronal VLCFA metabolism proceeds through a complex set of biochemical reactions responsible for their synthesis and degradation. VLCFAs are synthesized through a process of elongation which involves the addition of two-carbon units to existing fatty acyl chains in the ER. Specifically, there are two main steps in VLCFA synthesis: first, activation, which begins with activation of the initial fatty acid by attaching it to coenzyme A (CoA) to form palmitoyl-CoA, and second, elongation, where enzymes called elongases belonging to the ELOVL gene family^127^^,^^128^ catalyze the addition of two-carbon units (acetyl-CoA) to the growing fatty acid chain. These units are added sequentially, extending the fatty acid chain length. Various elongases are involved in the elongation process, each adding specific carbon units to a chain length beyond 22 carbons.

*Degradation of VLCFAs*

VLCFAs are degraded via β-oxidation, particularly in the peroxisome. In peroxisomal β-oxidation, VLCFAs undergo a stepwise oxidation process, releasing acetyl-CoA units and generating reducing equivalents (NADH and FADH_2_). The peroxisomal β-oxidation pathway is a four-step pathway involving: (1) oxidation, in which a series of enzymatic reactions catalyzed by acyl-CoA oxidase (ACOX) convert VLCFAs into 2-enoyl-CoA molecules which enter into the process of (2) hydration, to form 3-hydroxyacyl-CoA by the enzyme enoyl-CoA hydratase, (3) oxidation, where the 3-hydroxyacyl-CoA is further oxidized by the enzyme 3-hydroxyacyl-CoA dehydrogenase and is converted into 3-ketoacyl-CoA, and finally (4) thiolysis, where the 3-ketoacyl-CoA is broken down by thiolase to produce acetyl-CoA units and shortened acyl-CoA, which enters mitochondria for further oxidation. However, NADH and FADH_2_ are not transported to mitochondria, instead being reoxidized to NAD^+^ and FAD, respectively, to maintain the redox potential of peroxisomes.^129^^,^^130^

Genetic and clinical studies have highlighted the role of lipid metabolism in neuroinflammation.^6^^,^^20^^,^^38^ These discovery efforts have been particularly helped by the identification of mutations in specific genes that act as powerful stimuli for neuroinflammation. The discovery of *ABCD1* mutations^34^^,^^38^^,^^39^^,^^40^ in patients with X-linked adrenoleukodystrophy (X-ALD) revealed disrupted breakdown and consequent VLCFA accumulation,^39^^,^^40^^,^^41^^,^^42^^,^^43^^,^^44^ leading to oxidative stress and neuroinflammation in brain white matter. Since then, several other monogenic and other complex neuronal disorders have been associated with VLCFA metabolism (see Box 1).

### Composition and fluidity of neuronal cell membranes

VLCFAs are components of neuronal cell membranes,^45^^,^^46^ contributing to membrane fluidity and stability and influencing their biophysical properties. By doing so, they ensure the proper functioning of ion channels, receptors, and transporters. Therefore, alterations in membrane composition through changes in VLCFA levels could adversely affect these functions.

### Formation and maintenance of myelin sheath

Myelin is a lipid-rich sheath composed of 75%–80% lipids, nearly 20% of associated proteins, and water.^36^^,^^47^ It is produced by glial cells, including oligodendrocytes in the CNS and Schwann cells in the PNS, and insulates axons that facilitate efficient signal transmission in neurons. Lipids in the myelin sheath contain high levels of saturated VLCFAs including galactosylceramides, sphingomyelin, phosphatidylethanolamine, phosphatidylcholines, sulfatides, and others,^37^^,^^48^ which reduce myelin fluidity and establish a robust permeability barrier, effectively preventing ion leakage.^4^^,^^5^ In *Drosophila*, although glia lack myelin, the wrapping glia (see Figure 2) that insulate axons quite resemble Schwann cells^20^ and are rich in ceramide phosphoethanolamine (CPE), a lipid similar to sphingomyelin.^32^

In the white matter of the human brain, approximately half of the fatty acids in sphingomyelin are VLCFAs. Moreover, sphingomyelin of nervous tissue mainly contains stearic (18:0), lignoceric (24:0), and nervonic (24:1) acids. The composition of sphingomyelin in cerebral white matter undergoes significant changes during the first 2 years of human postnatal development. The proportion of 18:0 fatty acids decreased from 82% to 33% and the proportion of 24:1 fatty acids increased from 4% to 33%. This shift reflects a reduction in MCFAs and a corresponding increase in VLCFAs.^49^ The cerebrosides of the salamander spinal cord are also enriched for VLCFAs (>C27),^50^ as are frog brain and spinal cord sulfatides.^50^

Ceramide synthase 2 is mostly involved in the synthesis of VL-ceramides via esterification of VLCFAs into the sphingosine moieties. Evidence from ceramide synthase 2 knockout mice suggests that VLCFAs play a crucial role in myelin formation. These knockout mice harbor myelin with ceramide species containing SCFAs and show progressive loss of CNS and PNS myelin from early adulthood.^51^ Conversely, when VLCFAs or their by-products accumulate, they contribute to neuroinflammation and its adverse consequences, including the development of neurological disorders. The consequential inflammatory response has been implicated in compromising cognitive function. These findings underscore the intricate relationship between aberrant lipid metabolism and neuroinflammation, as well as the potential for cognitive impairment associated with such lipid abnormalities. Further supporting this concept, mice deficient in VLCFA esterification into ceramides exhibits significant myelin defects.^51^^,^^52^

### Neuronal development

VLCFAs play a role in neuronal development, particularly in axonal growth, dendritic branching, and axonal guidance.^31^ They exert neuroprotective effects under certain conditions, such as by reducing inflammation and promoting tissue repair. A mouse study showed that loss of GPSN2, a VLC-enoyl-CoA reductase involved in VLCFA synthesis, resulted in abnormal neural network development due to a defect in neuronal polarity determination.^31^ This led to disruption of VL-ceramide synthesis and affected phenotypes, which were rescued by C24:0 ceramide.^31^ A clinical study of patients with X-ALD revealed that loss of function of the ABCD transporter impairs transport of VLCFAs into the peroxisome and causes neuronal death, abnormal neural circuits and neurite growth, reduced neuron size, and defective axon and dendrite formation.^53^ Hence, the metabolism of VLCFAs is indispensable for safeguarding the structural and functional integrity of developing neurons.

## Mechanisms of VLCFA-mediated neuroinflammation

### VLCFA-associated cellular responses

VLCFAs, especially when overproduced, can evoke immune responses by activating resident immune cells in the brain,^54^ leading to chronic inflammation and neuronal damage. They can influence the function of immune cells, including microglia and immune cells from the bloodstream.^6^^,^^20^^,^^48^ Dysregulation of VLCFA metabolism potentially impacts microglial activation, migration, and cytokine production, which in turn affect the severity and duration of neuroinflammation.

Recent reports suggest that aging or demyelination leads to the VLCFAs accumulation in insulating glia in *Drosophila* and oligodendrocytes in humans. VLCFAs are converted to S1P, and excess S1P is transported to the corresponding insulated axons, where it triggers neuroinflammation by activating the NF-κB pathway in vertebrates (see Figure 1) and the IMD pathway in *Drosophila* (see Figure 2) and recruits macrophages into the CNS.^6^ S1P synthesis in response to CNS injury can also trigger neuroinflammation by activating cell surface receptors on astrocytes and increasing nuclear translocation of NF-κB.^55^

### VLCFA-induced oxidative stress

Oxidative stress is known to contribute to inflammation by activating inflammatory signaling pathways and promoting the release of proinflammatory molecules. Dysregulated VLCFA metabolism can increase oxidative stress and lipid peroxidation. The interplay between VLCFA metabolism and oxidative stress is well-illustrated in Mitchell syndrome, which is caused by a gain-of-function mutation (p.N237S) in *ACOX1*^20^ which is discussed in next section.

Mutations in peroxisomal *ABCD1* lead to VLCFA accumulation in the cytosol, plasma membranes, myelin, and lipid rafts of oligodendrocytes, astrocytes, microglia, and neurons, preventing their delivery to the peroxisome for degradation and increasing mitochondrial ROS and oxidative stress.^43^ Both ROS and accumulated VLCFAs directly interfere with the GSK3β-NRF2 pathway,^11^ and ROS also activate the NF-κB pathway to promote neuroinflammation. Microglia and astrocytes exposed to high VLCFA levels become activated and secrete inflammatory mediators such as TNF-α and IL-1β, amplifying tissue injury.^11^ Elevated ROS can also affect antioxidant systems as well as inflammatory pathways, leading to ER stress and demyelination, axonal degradation, and possibly neuronal death.^56^

Despite major advances, key aspects of *ABCD1*-related disease remain unresolved. It is still unclear why some patients develop childhood cerebral ALD (rapid inflammatory demyelination) while others develop AMN (slow axonopathy), even with identical mutations. Although elevated VLCFA levels are observed across all ALD subtypes, some individuals remain asymptomatic despite biochemical abnormalities. The underlying mechanisms of demyelination in X-ALD are thought to involve oxidative stress and neuroinflammation^42^; however, therapeutic interventions targeting these pathways, including antioxidants and immunosuppressants, have demonstrated limited efficacy. The precise molecular triggers that convert VLCFA accumulation into runaway neuroinflammation are not fully established, including the specific receptors or danger signals sensed by microglia.

## Application of *Drosophila* models in lipid metabolism research

### Historical overview

**Figure 3:**
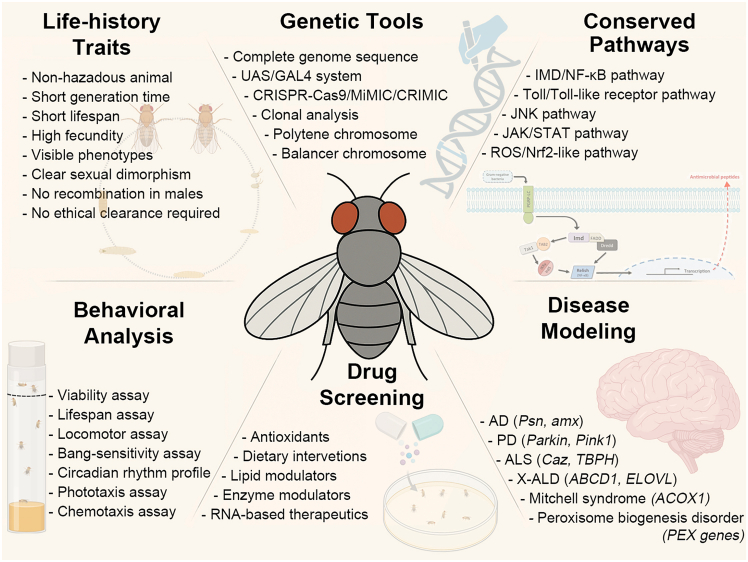
Features enabling *Drosophila* to model neurological disease linked to lipid metabolism The schematic highlights key attributes of the fly model: (1) favorable life-history traits enabling rapid, large-scale experimentation; (2) extensive genetic tools for precise perturbation of lipid-metabolism and neuronal genes; (3) conservation of major signaling pathways relevant to lipid homeostasis and neurodegeneration; (4) robust platforms for modeling human disease genes and mutations; (5) suitability for high-throughput *in vivo* drug screening; and (6) quantitative behavioral assays that provide functional readouts of neurological impairment and rescue. This figure was created with the help of BioRender.

For over a century, *Drosophila melanogaster* (fruit fly) has been demonstrated to be an excellent model organism to understand and explore numerous cellular processes and pathways relevant to human diseases. Since Morgan’s pioneering work in the early 1900s established *Drosophila* as a genetically tractable system, successive generations of researchers have leveraged its short life cycle, robust genetics, and highly conserved molecular pathways (Table 1; Figure 3) to model complex human disorders. Despite its biological simplicity, the deep evolutionary conservation of neuronal and glial functions, allows mechanisms related to innate immunity, neuronal integrity, and metabolic homeostasis to be interrogated with a level of genetic precision difficult to achieve in vertebrate systems. Despite the structural and anatomical distinctions between “lower” organisms and mammals (e.g., mice and humans have glial cells, but *Drosophila* has wrapping glial cells),^6^^,^^20^ these models have been valuable for studying the cellular and molecular mechanisms of neuroinflammation and associated neurodegenerative diseases.

**Table 1:** Advantages of using *Drosophila* as model organism

Whole-organism characteristics	Short generation time of 10 days, lifespan of 50–60 days, and high fecundity rate, providing a large pool of offspring at the desired developmental stage
	Males and females can be differentiated at different developmental stages to enable easy and quick screening based on sex
Genetic	Absence of meiotic recombination in males and availability of balancer chromosomes help to maintain mutant alleles and transgenes in desired combinations and the design of crosses to obtain desired genotypes
Phenotypic	*Drosophila* model offers direct visualization of gene expression patterns in different genetic and environmental conditions through its polytene chromosomes
	High degree of genomic sequence conservation
	Availability of a complete mapped genome sequence^57^
Experimental	Ability to implement powerful genetic tools like the UAS-GAL4 system^58^ to obtain targeted expression of desired transgenes and MARCM (mosaic analysis with a repressible cell marker) to generate somatic recombinant clones to create cells of different genotypes from a common parental cell^59^^,^^60^
	Simple activities, such as coordinated movement, larval food crawling, and climbing behavior, make it easy to detect defects^61^^,^^62^
CNS-related	Simple yet well characterized, vertebrate-like neural system in *Drosophila* contains sensory modalities such as taste, vision, olfaction, hearing, and heat sensation together with a shared cellular and molecular mechanism of learning and memory^63^^,^^64^
	Glial cells in *Drosophila* appear to have similar properties to those of humans,^65^ and the wrapping glia present in *Drosophila* are functionally similar to Schwann cells^6^^,^^20^
	*Drosophila* behaviors such as lifespan, locomotion, susceptibility to seizures, learning, memory, vision, and responses to stimuli can also easily be assayed to study the impact of genetic manipulations
	*Drosophila* allows rapid and cost-effective screening and validation of compounds and biomarkers compared with rodent-based models^66^^,^^67^
	Although the acquired immune system is absent, the innate immune system can serve as substitutes for investigating different facets of neuroinflammation
Ethical	The utilization of this model has the potential to mitigate certain ethical considerations that are commonly linked with that of vertebrate models

### Drosophila as a genetic tool

The availability of a fully sequenced and well-annotated *Drosophila* genome provides a strong foundation for precise genetic manipulation in neuroscience research. Central to this is the UAS/GAL4 system,^58^ which enables cell-type specific expression or silencing of genes, allowing detailed functional analysis of neuronal circuits. Genome-editing tools such as CRISPR-Cas9, MiMIC, and CRIMIC facilitate targeted gene knockouts, knock-ins, and reporter insertions, providing mechanistic insights into neuronal function and disease. Clonal analysis techniques, including MARCM,^59^^,^^60^ allow the study of mutant cell populations in otherwise wild-type tissues, while polytene chromosomes offer a cytogenetic map for visualizing gene arrangements and chromosomal alterations. Balancer chromosomes are indispensable for maintaining lethal or deleterious mutations across generations, ensuring the stable propagation of genetic lines. Together, these resources and tools make *Drosophila* an exceptionally tractable system for dissecting the genetic underpinnings of neural development, function, and pathology.

### Conservation of lipid metabolism pathways

VLCFA metabolism is highly conserved in *Drosophila melanogaster*, making it a robust model for investigating human neurodegenerative disorders (see Table 2; Figure 3). In mammals, elongases such as ELOVL1/4 catalyze chain-elongation steps to produce VLCFAs. In flies, elongase orthologs, elof, bond, and elo68 family members, perform analogous roles in generating VLCFAs that are incorporated into complex lipids. These lipids are degraded by peroxisomal β-oxidation, mediated by enzymes including Acox1 (dACOX1) and dependent on transporters such as ABCD1 and peroxisomal biogenesis proteins (PEX family).^70^^,^^77^ Disruption of these pathways including bubblegum (bgm) and double bubble (dbb) mutants,^68^ orthologs of human ACSL4/6, results in VLCFA accumulation, oxidative stress, locomotor impairments, and neurodegeneration, resemble human X-ALD and peroxisomal disorders. Downstream sphingolipid metabolism is also conserved: VLCFAs feed into ceramide synthesis via Schlank (ortholog of CERS), followed by hydrolysis by *CDase* (ortholog of ASAH2),^74^ phosphorylation by SK1/2 (ortholog of SPHK2/1), and degradation through S1P lyase (Sply, ortholog of SGPL1), generating bioactive lipids that can modulate immune signaling.^6^

**Table 2:** Conserved genes involved in VLCFA metabolism in human and *Drosophila*

Human Gene	Pathway step	*Drosophila* Ortholog/s	Insights from fly model
ELOVL1, ELOVL4, ELOVL6	VLCFA elongation	*eloF*, *bond*, *elo68α/β/γ*	Glial degeneration; VLCFA accumulation; activation of IMD pathway via ceramide to S1P conversion; neuroinflammation^6^
ACSL4, ACSL6	VLCFA activation (acyl-CoA synthetases)	*Bgm*, *dbb*	LCFA/VLCFA activation; neuroprotective role^68^
ABCD1, ABCD2, ABCD3	VLCFA transport into peroxisomes	*ABCD*, *CG2316*, *pmp70*	VLCFA accumulation; locomotor defects; oxidative stress sensitivity^69^
ACOX1, ACOX2, ACOX3	Peroxisomal β-oxidation (1^st^ step)	*CG5009*, *Acox57D-d/p*, *CG9527*	Glial phagocytic defects; LD accumulation; neuronal loss; both LOF and GOF cause degeneration^20^
PEX1, PEX6, PEX10, PEX12	Peroxisome biogenesis	*Pex1*, *Pex6*, *Pex10*, *Pex12*	Reduced peroxisome numbers; altered lipid profiles; neurodegeneration; male sterility; metabolic stress sensitivity^70^^,^^71^^,^^72^
CerS2	VL-ceramide synthesis	*Schlank*	Functions as a transcription factor repressing lipases to maintain fat storage^73^
ASAH2	VLCFA degradation	*CDase*	Regulates photoreceptor survival by maintaining ceramide balance, supporting rhodopsin endocytosis, and preventing apoptosis^74^
SPHK1, SPHK2	Convert sphingosine to S1P	*Sk2, sk1*	sk1 mutants lack S1P and accumulate sphingosine, causing metabolic sphingolipid imbalance^75^
S1P lyase (SGPL1)	S1P degradation	*Sply*	S1P accumulation; neurodegeneration; defective neuroinflammatory signaling^6^
PLA2G6	VLCFA-phospholipid remodeling	*iPLA2-VIA*	Mitochondrial defects; locomotor phenotypes; neurodegeneration resembling PLAN^76^

(orthologs of the corresponding human genes)

Accumulation of VLCFAs and VLCFA-containing sphingolipids in flies triggers robust neuroinflammatory responses through conserved signaling pathways analogous to mammals. The IMD pathway, a TNF/NF-κB analog, is activated in glia by sphingolipid perturbations, driving Relish-dependent antimicrobial peptide expression and inflammation.^6^ The Toll pathway similarly responds to stress and misfolded proteins via Dorsal/Dif, promoting glial immune activation. Stress-activated JNK and ROS/Nrf2 pathways link oxidative stress and mitochondrial dysfunction to inflammation, while JAK/STAT and Ca^2+^-dependent NFAT signaling regulate cytokine-like responses and glial proliferation. Together, these pathways demonstrate that VLCFA and sphingolipid metabolism are tightly coupled to conserved neuroinflammatory networks in *Drosophila*, providing a genetically tractable platform to dissect molecular mechanisms underlying lipid-driven neurodegeneration and immune activation relevant to human neurological disease.

### Insights from disease models in Drosophila

*Drosophila* is widely used as a model organism in neurobiological research (Table 1), particularly for studying CNS diseases and various neurological phenotypes. With respect to lipid metabolism, most human genes and metabolic pathways associated with lipid metabolism and CNS diseases are conserved in *Drosophila,*^63^ and given that it is possible to manipulate specific genes in nearly all cell types in *Drosophila*. This has allowed sophisticated manipulation of neuronal and glial cell populations to study their functional genomics and interactions.^6^^,^^8^ As such, *Drosophila* has been used to model various human disorders affecting the CNS such as AD, PD, ALS, X-ALD, Mitchell syndrome, peroxisomal disorders, fragile X syndrome, and ASD (see Figure 3).^20^^,^^78^ These models also allow for tracking the impact of genetic perturbations in one cell type on another. For instance, disruptions in lipid metabolism within glial cells can have consequences that extend beyond the glia, affecting neurons.^6^ This facilitates the investigation of complex multifactorial diseases such as AD and PD, characterized by interactions between different cell types within a living organism.

ACOX1 loss-of-function disrupts peroxisomal β-oxidation, causing toxic accumulation of VLCFAs in glial cells. The excess VLCFAs become incorporated into complex membrane lipids, altering membrane fluidity, lipid-raft organization, and overall structural stability. As a result, wrapping glia loses their ability to properly ensheathe the axons, leading to glial and axonal degeneration, impaired synaptic signaling, vision decline, motor deficits, and reduced lifespan.^20^ VLCFA overload also limits LCFA production, compromising glial energy metabolism and diminishing metabolic support to axons, thereby accelerating axonal loss. Lowering the VLCFA synthesis through bezafibrate or ELOVL1 inhibition restores survival and neurological function, confirming that VLCFA toxicity is the principal pathogenic mechanism.^20^ Although VLCFA buildup clearly damages glia, several mechanistic details remain unresolved. It is unknown how exactly VLCFA enrichment alters membrane biophysics and how much of the pathology is due to structural membrane disruption versus metabolic failure from loss of LCFA production. The discrepancy between severe human/fly phenotypes and relatively mild mouse phenotypes also remains unexplained, raising the possibility of species-specific compensation by ACOX2/3 or alternative β-oxidation pathways. Also, the downstream signaling pathways by which VLCFA-stressed glia initiate degeneration are still not fully defined.

Mitchell syndrome is one among the disorders modeled in *Drosophila*, where the gain-of-function dACOX1 p.N250S variant (corresponding to the hACOX1 p.N237S) enhances disease severity by stabilizing the enzyme’s conformation by forming active dimers in insulating glia, which dramatically increases peroxisomal β-oxidation activity without elevating VLCFAs. It generates excessive ROS, overwhelming antioxidant defenses in wrapping glia in *Drosophila* and Schwann cells in mice.^20^ This oxidative burden causes membrane peroxidation, mitochondrial impairment, and activation of apoptotic pathways, leading to pronounced glial degeneration, demyelination, progressive neuropathy, hearing loss, motor impairment, and ultimately axonal loss. Antioxidant intervention (e.g., N-acetyl cysteine amide [NACA] or catalase overexpression strongly suppresses degeneration, confirming that ROS excess is the main pathogenic mechanism in the gain-of-function state.^20^ Despite strong evidence for ROS-driven toxicity, key mechanistic questions remain. It is unknown which specific oxidative-stress pathways are responsible for Schwann cell death, such as mitochondrial apoptosis, ER stress, inflammatory responses, or peroxisome-mitochondria crosstalk.

Mutations in acyl-CoA synthetases (ACSLs) such as *bgm* and *dbb* in *Drosophila* impair the activation of VLCFAs to their CoA-esterified forms. This leads to the intracellular accumulation of VLCFAs, particularly C24:1 and C26:1, which disrupt membrane integrity, generate toxic lipid metabolites, and destabilize mitochondria.^68^ These lipid-driven insults cause degeneration of both neurons and their supporting glial/pigment cells, producing thinning and breakdown of the fenestrated membrane, disorganization of retinal and laminal architecture, and the appearance of granular inclusions and lytic cell death.^68^ The combined neuronal and glial degeneration ultimately manifests as progressive neurodegeneration and behavioral decline, mechanistically mirroring the pathology observed in X-ALD and leukodystrophies linked to ACSL dysfunction.

Mutant phenotypes of *Pex* (genes involved in peroxisome biogenesis) and other peroxisomal genes in *Drosophila* suggest their peroxisomal metabolism is comparable to mammals. According to several research studies, *Pex* gene mutations in *Drosophila* demonstrate that peroxisomes play a crucial role in VLCFA metabolism. These mutations lead to various phenotypic effects, including reduced peroxisome numbers, altered lipid levels,^70^ and increased sensitivity to glucose deprivation,^71^ and male sterility.^72^

As noted above, lipid metabolism and signaling play an important role in regulating neuroinflammation. Although lipid synthesis pathways such as lipogenesis, ketogenesis, and cholesterol synthesis and the β-oxidation pathway were discovered in humans, studies in *Drosophila* have led to new discoveries about lipid metabolism in CNS disorders.

Further investigations using *Drosophila* have also shown how mutations in the lipid metabolism gene *ELOVL* stimulate immune pathways to induce neuroinflammation^6^ through a novel mechanism whereby ceramide is converted into S1P to activate the IMD pathway (see Figure 2) leading to inflammation.^6^

### Drug screening and translational applications

One of the most impactful advantages of *Drosophila* is its capacity for rapid *in vivo* drug screening. The short generation time, low cost, and well-defined phenotypes enable efficient testing of therapeutic molecules across hundreds of animals simultaneously. Compounds such as NACA and bezafibrate have been identified using *Drosophila* models of ACOX1 and ELOVL dysfunction, where they ameliorate oxidative stress, restore glial and neuronal integrity, and rescue survival phenotypes.^6^^,^^20^ Additional classes of therapeutics including antioxidants, metabolic cofactors, peroxisome-boosting compounds, and gene therapy constructs have also been evaluated in flies, demonstrating the model’s power for preclinical triage of candidate molecules.

### Quantitative behavioral assays

*Drosophila* provides a diverse array of behavioral assays that are invaluable for probing neural function and dysfunction. Viability and lifespan assays offer fundamental readouts of organismal health and the impact of genetic or environmental manipulations on survival. Locomotor assays, including negative geotaxis and climbing tests, quantify motor coordination and age-dependent neurodegeneration. Bang-sensitivity assays measure seizure-like activity in response to mechanical stress, providing a model for epilepsy research. Circadian rhythm profiling allows assessment of endogenous biological clocks and their neural regulation. Simple yet informative sensory assays, such as phototaxis and chemotaxis, evaluate visual and olfactory-guided behaviors, respectively, enabling insights into neural circuitry and cognitive function. Collectively, these behavioral paradigms offer a robust toolkit to link genetic and cellular perturbations with functional outcomes in the nervous system.

## Exploiting lipid metabolism for therapeutic benefit

The preceding discussion highlights that targeting VLCFA metabolism has potential as a therapeutic approach for a variety of disorders, including those involving neuroinflammation and neurodegeneration. While research is still ongoing, and specific therapies may differ depending on the context, we present several broad approaches to targeting VLCFA metabolism.

### Enzyme inhibition or activation

Developing drugs that target enzymes involved in VLCFA metabolism, such as elongases (ELOVLs), and enzymes involved in peroxisomal β-oxidation, such as ACOX1, dehydrogenases, and thiolases, could potentially regulate VLCFA synthesis and breakdown, thereby influencing disease-related phenotypes. Both loss-of-function and gain-of-function mutations in *ACOX1* are associated with axonal loss. NACA, an antioxidant, mitigates the detrimental effects of ROS generated by gain-of-function mutations. Although NACA is highly effective in experimental models, it is not yet clinically available for patient use.

Moreover, some lipid-lowering drugs, such as fibrates, have been investigated for their potential to modulate lipid metabolism, including VLCFA levels. For example, bezafibrate, a PPAR agonist, inhibits ELOVL1 and reduces fatty acid synthesis via direct inhibition of fatty acid elongation.^79^^,^^80^ Fingolimod, an S1P receptor modulator,^6^^,^^81^ is used to treat MS. It strongly ameliorates the phenotypes caused by excess VLCFAs by affecting the immune system to reduce the inflammation associated with MS in a murine experimental autoimmune encephalomyelitis model.^6^

Pyrazole amides and pyrimidine ether-based compounds are effective ELOVL1 inhibitors *in vitro* in patient-derived X-ALD cells by reducing C26:0 VLCFA production. They also downregulate C26:0 VLCFA levels in X-ALD mice, reaching levels comparable to those in healthy mice, particularly in the brain. Therefore, these ELOVL1 inhibitors could be a promising means to restore basal VLCFA levels in patients with X-ALD.^41^^,^^82^

Acetyl-CoA carboxylase (ACC), a key enzyme in lipid synthesis with two isoforms, ACC1 (cytoplasmic) and ACC2 (mitochondrial), catalyzes the production of malonyl-CoA from acetyl-CoA.^83^ Depletion of ACC1 significantly decreases VLCFA levels^84^ without affecting LCFA levels. Inhibitors like ND-630 from Nimbus Therapeutics, which disrupts ACC subunit dimerization, prevent the conversion of acetyl-CoA to malonyl-CoA.^85^^,^^86^ Meanwhile, Pfizer’s PF-05221304 binds to the C-terminal domain of ACC and is currently in phase II clinical trial to reduce inflammation via *de novo* lipogenesis in non-alcoholic fatty liver disease.^87^ Therefore, inhibition of ACC could be a potential target to reduce VLCFA levels.

### Gene therapy

Gene therapy involves the direct introduction of functional genes crucial for specific conditions to restore typical cellular function. Lipid metabolism could potentially be regulated by altering gene expression, which can be achieved using various approaches including small molecules, RNA interference (RNAi), epigenetic modifiers, and gene editing techniques (e.g., CRISPR/Cas, transcription activator-like effector nucleases [TALENs]).^88^ Adeno-associated virus (AAV) vectors are widely used for gene therapy because they express the desired genes efficiently and consistently, have a low risk of causing an immune response, and are easy to adjust. AAVs can also be used to specifically target the CNS.^89^

AAV-based treatments have shown promise in lipoprotein lipase (LPL) deficiency, a serious lipid metabolism disorder. UniQure’s Glybera uses an AAV1 vector to deliver a gain-of-function *LPL* variant (S447X) directly into muscles, thereby restoring LPL activity and mitigating the complications associated with the deficiency.^90^ ABCD1, a peroxisomal membrane transporter, moves VLCFAs from the cytoplasm to peroxisomes.^91^ AAV9 has been used to deliver *ABCD1* to the nervous system and adrenal gland *in vitro* and *in vivo*, setting the scene for reducing VLCFAs.^92^ Clinically, Skysona, approved for early-stage patients with X-ALD harboring the *ABCD1* mutation, employs Lenti-D lentiviral vector transduction *in vitro* to introduce a functional *ABCD1* gene copy into the patient’s hematopoietic stem cells as a one-time gene therapy.^41^^,^^93^^,^^94^

Patients with very long-chain acyl-coA dehydrogenase deficiency (VLCADD) lack VLCAD, which is crucial for fatty acid oxidation in mitochondria, leading to energy reduction and accumulation of long-chain metabolites. Mice with VLCADD cannot maintain their body temperature when exposed to cold. However, AAV9-VLCAD administered to VLCADD mice significantly decreased the accumulation of LCFAs, indicating therapeutic potential.^95^ Since patients with VLCADD have an increase in circulating inflammatory mediators,^33^ targeting the *VLCAD* gene might also regulate neuroinflammation.

### Nutritional modification

Dietary choices significantly affect both physical and mental health. Dysregulation of lipid metabolism is also associated with obesity.^96^^,^^97^^,^^98^ Excessive intake of high-fat foods can lead to increased levels of circulating lipids, which can contribute to lipid accumulation in the brain. The accumulation may play a role in the development of insulin resistance, which impairs the uptake and utilization of lipids in various regions of the brain. Consequently, it can disrupt signaling, promote neuroinflammation,^99^ and ultimately lead to neuronal dysfunction.^96^^,^^97^ Therefore, dietary choices significantly impact not just physical well-being, but also brain health. However, the precise relationship between obesity and the involvement of specific lipids, particularly VLCFAs, remains to be elucidated and requires further clarification.

Cellular LCFAs, precursors to VLCFAs, are available through *de novo* synthesis, triglyceride hydrolysis, or dietary intake. As the brain synthesizes only a limited number of fatty acids, the majority, including LCFAs, must be acquired from the bloodstream,^100^^,^^101^ usually from dietary sources rather than through *de novo* synthesis and modification.^102^^,^^103^ Dietary fatty acids can enter the brain through several pathways. Of these, LCFAs utilize specific transporters such as fatty acid transporter protein 1 (FATP-1) and fatty acid translocase (FAT)/CD36.^103^ There is also ongoing debate about whether VLCFAs can cross the BBB. Some studies have suggested that controlling the dietary intake of VLCFAs does not impact central VLCFA levels as large lipid species cannot cross the BBB in either direction.^48^^,^^104^ Conversely, others have proposed that both LCFAs and VLCFAs can cross the BBB through FATPs and FAT/CD36.^101^^,^^103^^,^^105^ Nonetheless, dietary regulation may have the potential to modulate VLCFA levels in the brain and treat associated disorders.

A more specific approach might be to adjust the consumption of specific fatty acids in the diet to impact VLCFA metabolism. Increasing intake of essential fatty acids or polyunsaturated fatty acids may influence VLCFA synthesis and incorporation into membranes. Furthermore, certain dietary supplements, such as antioxidants or essential fatty acid supplements, might support VLCFA metabolism and help mitigate oxidative stress associated with dysregulated metabolism. For example, docosahexaenoic acid (DHA) is a dietary supplement that could be used to control neuroinflammation.^106^ In mouse models, DHA can protect oligodendrocytes from mitochondrial dysfunction, oxidative stress, and autophagy caused by VLCFAs.^107^

## Concluding remarks and future perspectives

Investigating the cellular functions of specific lipids in disease states presents both exciting opportunities and significant challenges, due to the complexity of lipid metabolic pathways and the diversity of cell types within the brain. Lipids are increasingly recognized as central contributors to the pathobiology of CNS diseases through their essential roles in modulating neuroinflammation. This review focused on the role of lipids, especially VLCFAs, in maintaining brain health and disease. Perturbed VLCFA metabolism through specific gene defects often has a severe impact on lipid homeostasis, consequently promoting neuroinflammation and neurological disorders. Several studies have now consistently demonstrated the relationship between VLCFA accumulation and neurological disorders, although direct causative evidence is still lacking.

*Drosophila* has emerged as a uniquely powerful model system to bridge this gap. Its unparalleled genetic toolkit enables precise manipulation of lipid metabolic genes and rapid *in vivo* assessment of their functional consequences across conserved cellular pathways. These models allow researchers to dissect how perturbations in VLCFA metabolism influence neuronal physiology, glial responses, and organismal phenotypes, thereby linking molecular defects to disease-relevant outcomes. Mechanistic studies using fly models are accelerating the identification of signaling pathways underlying lipid-driven neurotoxicity and aiding the evaluation of candidate therapeutics, including antioxidants, small-molecule inhibitors, and gene-based approaches. However, several challenges remain unanswered. Critical gaps remain in our knowledge of the exact molecular mechanisms of lipid-mediated neurotoxicity and how imbalanced lipid homeostasis leads to peroxidation and neuronal death. Bridging these gaps by leveraging the strengths of *Drosophila* to model human lipidopathies with precision could facilitate the development of new therapeutics to regulate lipid homeostasis to prevent and treat neuronal disorders.

## Acknowledgments

H.-l.C. is supported by the 10.13039/100002558Warren Alpert Foundation. We acknowledge support from Mitchell Foundation and start-up fund from 10.13039/100015581Houston Methodist Academic Institute.

## Author contributions

H-L.C conceived and supervised the sutdy and led the writing of the manuscript. R.K.S prepared figures and drafted the initial version. Y.Y. contributed to writing and the vertebrate-related content. All authors reviewed and approved the final manuscript.
